# Selective Separation and Recovery of Cadmium from High-Concentration Zinc Smelting Dust Leachate via N235/TBP Solvent Extraction

**DOI:** 10.3390/ma19112368

**Published:** 2026-06-02

**Authors:** Kangwen Li, Xiaohua Yu, Qingfeng Shen, Gang Xie, Anming Xie

**Affiliations:** 1 School of Metallurgical and Energy Engineering, Kunming University of Science and Technology, Kunming 650093, China; 20232102052@stu.kust.edu.cn (K.L.); shenqf@kust.edu.cn (Q.S.); 2 State Key Laboratory of New Technologies for Metallurgical Processing of Non-Ferrous Metals, Kunming 650093, China; gangxie@sina.com; 3 Qinghai Coal Mine Design and Research Institute Co., Ltd., Xining 810001, China; 20252202131@stu.kust.edu.cn

**Keywords:** N235, solvent extraction, cadmium recovery, extraction mechanism, zinc smelting dust

## Abstract

**Highlights:**

- Developed a robust N235/TBP solvent extraction system to recover Cd from highly concentrated (44.55 g/L) zinc smelting dust leachate.
- Achieved 99.80% Cd extraction efficiency via a three-stage countercurrent process, realizing deep separation from As and Zn.
- Elucidated the anion-exchange mechanism via FT-IR and ESI-MS, confirming the formation of the (R_3_NH)_2_CdCl_4_ complex.
- Demonstrated that 15% (*v*/*v*) TBP effectively eliminates third-phase formation, optimizing organic phase viscosity and phase disengagement.

**Abstract:**

The efficient recovery of highly concentrated cadmium (44.55 g/L) from zinc smelting dust leachate is recognized as a significant metallurgical challenge. In this study, we focused on the selective separation of Cd from coexisting arsenic and zinc using trioctylamine (N235) as the extractant. Accordingly, key operational parameters including initial pH, extractant concentration, phase ratio, and temperature were optimized in a systematic manner. Under the optimized conditions of 30% N235, 15% TBP, and 55% sulfonated kerosene by volume, together with an initial pH of 0.5, an organic to aqueous phase ratio of 1 to 1, and a temperature of 20 °C, a three-stage countercurrent extraction process was found to dramatically enhance the Cd extraction efficiency to 99.80% while successfully rejecting As. Subsequently, stripping with 0.7 mol/L aqueous ammonia achieved an 81.4% stripping efficiency in a single stage, and washing with 1.0 mol/L HCl ensured complete regeneration of the organic solvent. Furthermore, Fourier transform infrared spectroscopy (FT-IR) and electrospray ionization mass spectrometry (ESI-MS) analyses corroborate that the extraction proceeds via an anion exchange mechanism. Specifically, within the chloride rich acidic environment, protonated N235 was shown to preferentially coordinate with the tetrachlorocadmate anion CdCl_4_^2−^ to form the highly stable and lipophilic complex (R_3_NH)_2_CdCl_4_. Overall, this work provides a scalable technological framework and a robust theoretical foundation for the extraction of highly concentrated heavy metals from complex secondary metallurgical resources.

## 1. Introduction

Cadmium (Cd) is a highly toxic yet technologically critical heavy metal that is found naturally at low concentrations in the Earth’s crust [1,2,3]. It is extensively utilized across a range of advanced technological fields, including high-performance electronics, nickel–cadmium batteries, and photovoltaic systems [4,5]. In nature, discrete economic minerals of cadmium are not formed [6,7,8]; instead, cadmium is intimately associated with polymetallic sulfide ores and is primarily recovered as an important by-product during the smelting of copper, lead, and zinc [9,10,11,12], as well as from electrolytic residues generated in these processes [13,14,15,16]. With the sustained and environmentally conscious development of the zinc smelting industry, the generation of cadmium-rich secondary resources, most notably smelting flue dust, has been substantially increased [17]. Consequently, the efficient extraction and recovery of high-concentration cadmium from zinc smelting flue dust leachates has been recognized as critically important, not only for resource conservation and the promotion of a circular economy but also for the mitigation of potential environmental hazards [18].

Conventional techniques employed for the recovery or separation of metal ions are generally classified into cementation, chemical precipitation [19,20], ion exchange, membrane separation [21,22], and solvent extraction [23,24]. However, ion exchange resins are susceptible to fouling and require frequent regeneration; membrane separation also suffers from membrane fouling and has a short lifespan [25]. Precipitation and solvent extraction are common methods for recovering high-concentration metals. The chemical precipitation involves adding precipitants (sulfides, hydroxides, etc.) to convert substances dissolved in water into insoluble salts for precipitation. Although neutralization precipitation is commonly applied in industrial practice, this method is frequently characterized by excessive reagent consumption and the generation of substantial volumes of hazardous solid waste, which inevitably results in serious secondary environmental pollution [26]. In contrast, solvent extraction has been increasingly recognized as one of the most effective methodologies, primarily owing to its relatively lower operational costs, high selectivity, and superior metal recovery efficiencies. These inherent advantages render solvent extraction particularly well suited for the separation of multiple metal species from complex aqueous matrices [27]. An evaluation of precipitation and extraction methods for cadmium recovery is provided in Table 1.

A wide variety of extractants, encompassing acidic [35,36], alkaline [37], neutral and synergistic extraction systems [38,39], have been systematically investigated for the recovery of cadmium. Despite this extensive research, a critical technical bottleneck is still encountered, as the majority of existing solvent extraction studies have been predominantly focused on the treatment of solutions containing relatively low concentrations of cadmium, typically in the range of 2 to 8 g/L [40,41,42,43,44,45,46,47,48,49]. When these conventional systems face the actual zinc smelting dust leaching solution (cadmium concentration exceeds 40 g/L and coexists with high concentrations of zinc and As), there will be problems such as emulsification of the organic phase, formation of a third phase, and a sudden drop of selectivity. Consequently, these conventional extraction systems are widely regarded as inadequate for the effective processing of actual leachates derived from zinc smelting flue dust. Such industrial solutions are characterized not only by exceptionally elevated cadmium concentrations but also by the co-existence of complex interfering ionic species.

To address this deficiency, an appropriate extractant for the recovery of high-concentration cadmium from smelting dust leachate is considered essential. Trioctylamine (N235), a conventional tertiary amine, is noted for its exceptional ability to remove anionic metal complexes from acidic environments. In this study, N235 was systematically employed to examine the extraction and separation of cadmium from high-concentration zinc smelting flue dust leachate. The extraction behavior, optimal operating parameters, and selective separation of arsenic and zinc were thoroughly assessed. The fundamental extraction mechanism was clarified through the combined application of Fourier-transform infrared spectroscopy (FT-IR) and electrospray ionization mass spectrometry (ESI-MS). This work is anticipated to provide a valuable technical reference and a robust theoretical foundation for the efficient recovery of high-concentration cadmium from analogous complex systems.

## 2. Materials and Methods

### 2.1. Experimental Materials

A synthetic solution containing 44.55 g/L Cd was prepared by dissolving analytically pure cadmium sulfate (CdSO_4_, Xilong Scientific Co., Ltd., Shantou, China) in deionized water, with an initial pH of 6.0. Actual cadmium-bearing smelting dust leachate was sourced from a zinc refinery in Yunnan Province, China, with an initial pH of 2.8 and metal contents of Cd 44.55 g/L, As 5.77 g/L, Zn 2.09 g/L, and Cl 110 mg/L. The primary extractant trioctylamine (N235, ≥99%), co-extractant tributyl phosphate (TBP, ≥98%), and diluent sulfonated kerosene were obtained from Chongqing Kangpu Chemical Co., Ltd., Chongqing, China. Hydrochloric acid and aqueous ammonia (~25%) were employed for pH adjustment and stripping. All reagents were used as received and without any further purification.

### 2.2. Solvent Extraction and Stripping Tests

Aqueous phase pH and chloride concentration were adjusted using HCl and NaCl prior to extraction. The adjusted solution was mixed with the organic phase (comprising N235, TBP, and sulfonated kerosene) at a fixed phase ratio in a conical flask and shaken in a thermostatic water bath for a set equilibration time. After phase disengagement in a separatory funnel, metal concentrations in the raffinate were analyzed to determine extraction efficiency.

Stripping was performed by contacting the Cd-loaded organic phase with aqueous ammonia under identical operational conditions. The stripped organic phase was subsequently washed with HCl for complete regeneration and recovery of residual cadmium.

### 2.3. Data Treatment

Metal concentrations in aqueous solutions were analyzed by a third-party professional testing mechanism with CNAS and CMA qualifications. In this analysis, Cd and Zn were determined by atomic absorption spectrophotometry, while As was determined by titration using a bromate standard solution with methyl orange as an indicator(the titration endpoint was the color change in the solution from red to colorless). Extraction mechanisms were investigated using FT-IR (Bruker Tensor 27, Bruker Optics GmbH & Co. KG, Ettlingen, Germany) and ESI-MS (Bruker ESI Q-TOF MS/MS).

Performance was evaluated via distribution ratio (*D*), extraction efficiency (*E*), stripping efficiency (*S*), and separation factor (*β*). *D* was defined as the organic-to-aqueous metal concentration ratio at equilibrium. *E* and *S* represented the respective percentages of metal transfer between phases. *β* was calculated as the quotient of distribution ratios for two metal species under identical conditions.

(1) $$D=\frac{C_{\mathrm{org}}}{C_{\mathrm{aq}}}$$

(2) $$E=(1-\frac{C_{aq}^{’}V_{aq}^{’}}{C_{aq}V_{aq}})\times 100\%$$

(3) $$E_{s}=\frac{M_{\mathrm{aq}}V_{sa}}{M_{\mathrm{org}}V_{so}}\times 100\%$$

(4) $$\beta =\frac{D_{A}}{D_{B}}$$

where *C_org_*, *C_aq_* and *C’_aq_* denote the concentrations of the extract in the loaded organic phase, extraction solution and raffinate, respectively; *V_aq_* and *V’_aq_* represent the volumes of the extraction solution and raffinate, respectively; *M_org_* and *M_aq_* stand for the concentrations of metal ions in the loaded organic phase prior to stripping and in the aqueous phase after stripping, respectively; *V_so_* and *V_sa_* denote the volumes of the loaded organic phase before stripping and the aqueous phase after stripping, respectively; and *D_A_* and *D_B_* refer to the distribution ratios of substances *A* and *B* under the same extraction conditions, respectively.

## 3. Results and Discussion

### 3.1. Experimental Study on the Extraction Conditions of Cd in Simulated Solution

#### 3.1.1. Effects of pH, Cl^−^ Concentration and Temperature

The influence of pH, Cl^-^ concentration, and temperature optimization on cadmium extraction was evaluated using a synthetic cadmium sulfate solution at 40% N235, O/A phase ratio of 1 to 1, and an oscillation time of 5 min.

**Figure 1:**
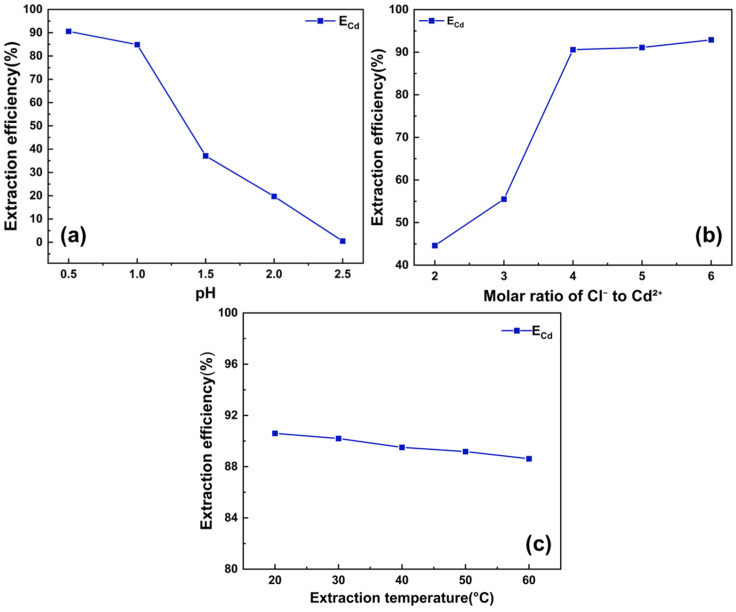
Effects of various parameters on the extraction of Cd in the simulated solution (**a**) initial pH (conditions: 25 °C and Cl^−^ to Cd^2+^ molar ratio 4 to 1); (**b**) Cl^−^ concentration(conditions: initial pH 0.5 and 25 °C); (**c**) temperature(conditions: initial pH of 0.5 and Cl^−^ to Cd^2+^ molar ratio 4 to 1).

As shown in Figure 1a, extraction efficiency was found to increase with decreasing pH. This behavior was attributed to the protonation of N235 to form the active (R_3_NH)^+^ cation and to enhanced formation of extractable cadmium chloro complexes under acidic conditions [50,51,52]. The extraction efficiency of cadmium was improved with an increasing Cl/Cd molar ratio. Extraction efficiency reached 90.6% at a ratio of 4 to 1, consistent with the stoichiometry of CdCl_4_^2−^ formation [53], and attained a maximum of 93.0% at a ratio of 6 to 1 (Figure 1b). The higher chloride dosage suppressed competing reactions involving protonated extractant and intermediate chloro complexes. Figure 1c illustrates that extraction efficiency diminished marginally with rising temperature, suggesting a feeble temperature dependence. A temperature of 20 °C was established for further trials.

#### 3.1.2. Effects of N235 Concentration, TBP Concentration and O/A Ratio

The influence of N235 concentration, TBP concentration, and O/A ratio on cadmium extraction was evaluated using a synthetic cadmium sulfate solution at 20 °C, initial pH 0.5, oscillation time of 5 min, and Cl to Cd molar ratio of 4 to 1.

**Figure 2:**
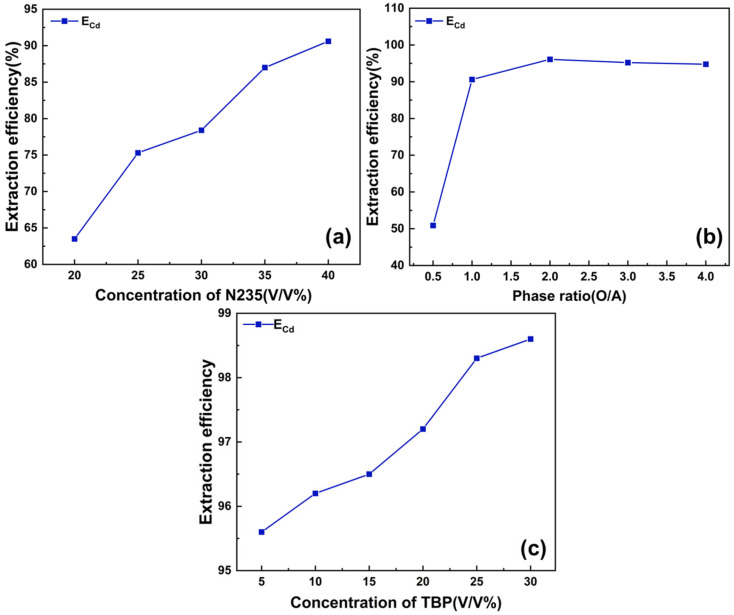
Effects of various parameters on the extraction of Cd in the simulated solution (**a**) N235 concentration(conditions: O/A 1 to 1); (**b**) (O/A)phase ratio(conditions: 40% N235): (**c**) TBP concentration(conditions:40% N235 and O/A 1 to 1).

As shown in Figure 2a, extraction efficiency increased progressively from 63% to 90.6% as the N235 concentration was raised from 20% to 40% by volume. Higher extractant concentrations were found to increase organic phase viscosity (pure N235 viscosity 8.41 mPa·s), thereby impairing phase contact and mass transfer [54]. A concentration of 40% by volume was therefore selected. When the O/A ratio was varied from 1:2 to 4:1, extraction efficiency increased from 50.9% to 96.1% (Figure 2b). An O/A ratio of 1 to 1 was chosen to balance extraction performance and solvent economy. With 40% N235 in sulfonated kerosene employed as the organic phase, a third intermediate layer was observed during extraction. The addition of tributyl phosphate (TBP) was investigated for the suppression of third phase formation. It was found that the third phase was effectively eliminated and phase disengagement was improved at a TBP concentration of 15% by volume (Figure 2c). This is because the P=O group of TBP was considered to form hydrogen bonds with the N–H moiety of (R_3_NH)^+^, thereby inhibiting water incorporation and reducing interfacial tension [55]. In addition, the extracted complex would have strong polarity, and self-aggregates to form reverse micelles in the non-polar diluent kerosene due to dipole interaction. When the aggregation is too much, the third phase is finally formed. With the addition of TBP, the order of molecular arrangement in the organic phase was changed. The three butyl chains in the molecular structure provided considerable steric hindrance, destroyed the close packing of polar aggregates, and regulated the microstructure and phase behavior of the whole organic phase [56,57]. It was also noted that TBP interacts with N235, increasing its solubility in kerosene, reducing organic phase viscosity, and enabling a greater number of R_3_N molecules to participate in the extraction process [58].

#### 3.1.3. The Influence of Competitive Ion Zn^2+^

**Figure 3:**
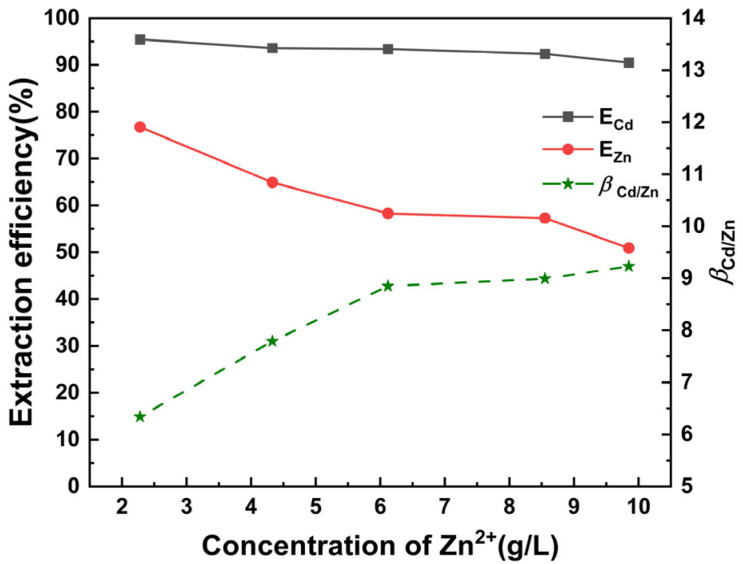
Influence of competing ions of Zn^2+^.

Because cadmium commonly coexists with zinc in smelting dust leachates, the influence of Zn(II) concentration on cadmium extraction selectivity was assessed. Under optimal conditions of 40% N235, 15% TBP, O/A 1 to 1, initial pH 0.5, 20 °C, and Cl to Cd molar ratio of 6 to 1, increasing Zn(II) concentration led to a gradual decline in cadmium extraction efficiency (Figure 3). This was ascribed to the competitive formation of zinc-chloro complexes (ZnCl^+^, ZnCl_2_, ZnCl^3−^, ZnCl_4_^2−^) which consume available amine salt cations [59].

Based on the systematic evaluation of the seven operational parameters described above, the optimal extraction conditions for cadmium recovery from synthetic sulfate solution were established. The organic phase composition was recommended to consist of 40% N235 and 15% TBP by volume in sulfonated kerosene. The aqueous phase was maintained at an initial pH of 0.5, and a chloride to cadmium molar ratio of 6 to 1 was employed. An organic to aqueous phase ratio of 1 to 1 and an operating temperature of 20 °C were adopted. Under these conditions, a cadmium extraction efficiency of 93.0% was consistently achieved in the synthetic system. Furthermore, the addition of 15% TBP was demonstrated to effectively eliminate third-phase formation and to enhance phase disengagement. The presence of co-existing zinc ions was found to exert a moderate competitive effect on cadmium extraction. Nevertheless, sufficient selectivity was retained by the N235/TBP system to warrant further investigation with authentic industrial leachate. These optimized parameters were therefore employed as the baseline for all subsequent experiments involving the actual zinc smelting flue dust leachate.

### 3.2. Extraction Mechanism Study

#### 3.2.1. Composition of Cadmium Complexes in the Chloride System

**Figure 4:**
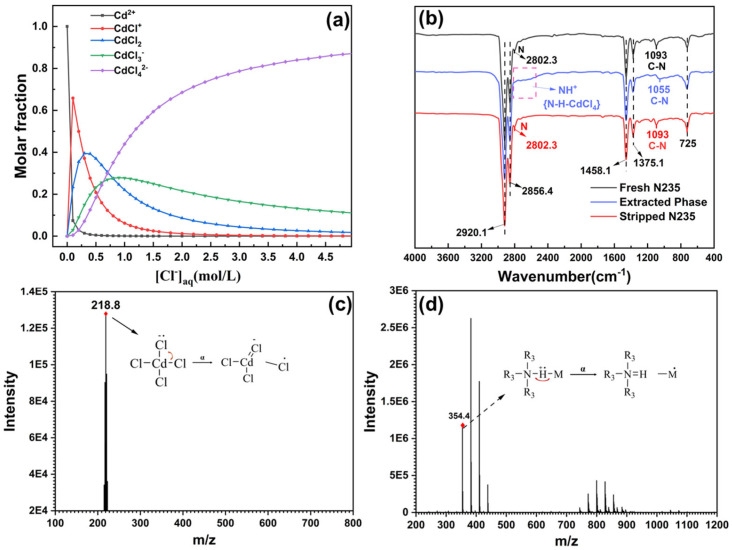
Extraction mechanism. (**a**) Mole fraction distribution; (**b**) FT-IR; (**c**,**d**) ESI-MS.

In weakly acidic systems containing elevated chloride ion concentrations, cadmium is present not as free Cd^2+^ cations, but predominantly as a series of chloro complexes of the general form CdCl_j_^2−j^, where j equals 1, 2, 3, or 4 [60]. From the cumulative formation constants [61], the molar fractions of the respective cadmium species at varying chloride concentrations were calculated (Figure 4a). As the Cl^−^ concentration was increased from 0.7 to 5.0 mol/L, the molar fractions of the cadmium chloride complexes in the solution followed the order: [CdCl_4_^2−^] > [CdCl_3_^−^] > [CdCl_2_] > [CdCl^+^] > [Cd^2+^].

Given that the experimental chloride concentration was maintained at approximately 2.4 mol/L, the CdCl_4_^2−^ species was unambiguously identified as the predominant form present in the aqueous phase. In accordance with the anion-exchange mechanism inherent to tertiary amine extractants, neutral or cationic species cannot be extracted. Between the available anionic species, CdCl_4_^2−^ is characterized by a larger thermochemical radius, a lower surface charge density, and a consequently weaker hydration shell in comparison with CdCl^3−^ [62]. These thermodynamic advantages were found to substantially reduce the energy barrier for phase transfer, thereby rendering CdCl_4_^2−^ the primary species selectively partitioned into the organic phase.

Considering the coexistence of cadmium and zinc in the subsequent dust leaching solution, a small amount of zinc ions will also be co-extracted with Cl^-^, but the presence of zinc ions has a relatively small effect on the extraction efficiency of cadmium. The reason lies in the combined effects of hydration and chlorocomplex formation. Since water is a hard Lewis base, Zn^2+^ (a borderline acid) is more strongly hydrated than Cd^2+^ (a soft acid), which stabilizes Zn^2+^ in the aqueous phase. In contrast, Cd^2+^, being less tightly hydrated, interacts more readily with Cl^−^ (an intermediate hard base according to HSAB theory) [63,64].

#### 3.2.2. FT-IR Analysis of Organic Phases

To elucidate the structural evolution of the extractant and the coordination environment, the FT-IR spectra of the fresh, Cd-loaded, and stripped organic phases were compared (Figure 4b). The characteristic aliphatic C–H bending vibrations, assigned to CH_3_ at 1375 cm^−1^ and CH_2_ at 1458 cm^−1^, remained entirely unshifted, thereby confirming that the hydrophobic alkyl chains were not directly involved in the coordination process [65]. Critically, the characteristic C–N stretching vibration observed at 1093 cm^−1^ in the fresh N235 [66] was found to undergo a red shift to 1055 cm^−1^ in the Cd-loaded phase. This pronounced shift confirmed the active participation of the nitrogen atom in complexation. Furthermore, the characteristic absorption band at 2802 cm^−1^, which was attributed to the protonated amine group R_3_NH^+^, exhibited marked attenuation in the loaded organic phase. This reduction in peak intensity was ascribed to the inherently weak absorption of the NH^+^ moiety [67], combined with the strong electrostatic interactions established between the R_3_NH^+^ cation and the bulky CdCl_4_^2−^ anion. Following the stripping process, the spectrum was observed to revert completely to that of the fresh organic phase, indicating that the metal–ligand bonds were successfully cleaved and demonstrating the excellent reversibility and recyclability of the N235 extractant.

#### 3.2.3. ESI-MS Analysis of Organic Phases

Electrospray ionization mass spectrometry (ESI-MS) was employed for the direct identification of the extracted species present in the loaded organic phase. In the negative-ion spectrum presented in Figure 4c, a prominent signal was detected at a mass-to-charge ratio (*m*/*z*) of 218.8. This signal was assigned to the CdCl^3−^ fragment, which is typically generated through the cleavage of a single Cd–Cl bond from the parent CdCl_4_^2−^ complex during the high-energy ionization process. In the corresponding positive-ion spectrum (Figure 4d), a distinct peak was observed at *m*/*z* 354.4. This peak was attributed to the intact protonated amine cation R_3_NH^+^, thereby verifying the successful protonation of the N235 extractant. The sequential mass differences of 28 units were consistent with the stepwise fragmentation of the alkyl chains.

**Figure 5:**
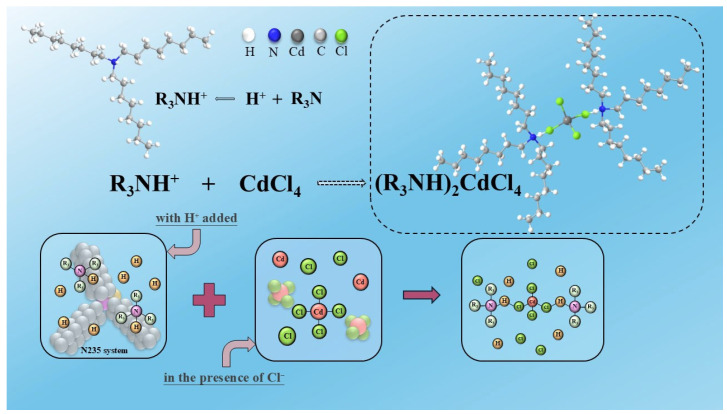
Mechanism diagram.

The comprehensive analysis of cadmium chloro complex speciation, together with FT-IR and ESI-MS results, revealed that two protonated amine molecules coordinate with one divalent cadmium chloro complex. In other words, two moles of protonated N235 cation react with one mole of aqueous cadmium ion and four moles of chloride ion to yield the neutral extracted complex (R_3_NH)_2_CdCl_4_ in the organic phase, and this reaction is schematically illustrated in Figure 5. This finding is consistent with the previous report by Sato et al. [68], who identified the same extracted complex (R_3_NH)_2_CdCl_4_ in the N235 extraction system for Cd(II).

### 3.3. Extraction Studies Using Actual Smelting Dust Leachate

Given that the composition of the actual smelting dust leachate is more complex than that of the synthetic cadmium sulfate solution, with multiple metal ions (e.g., As, Zn) and anions coexisting, the extraction performance of the N235 system for the actual leachate was validated under the same operating conditions previously optimized with the synthetic solution.

#### 3.3.1. Effect of Initial pH

**Figure 6:**
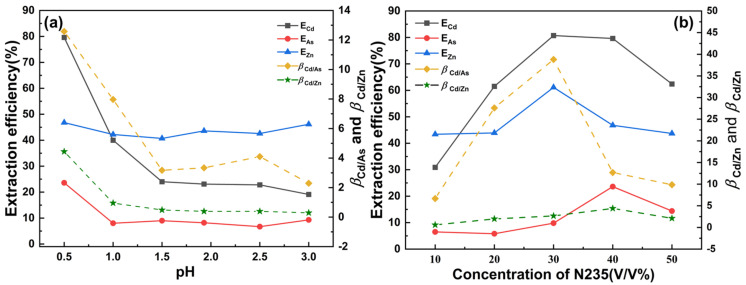
Effects of various parameters on the extraction of Cd in the smoke dust leaching solution (**a**) initial Ph; (**b**) extractant concentration.

To confirm the extraction procedure in conditions pertinent to industry, the actual Cd-bearing smelting dust leachate was utilized as the aqueous phase. We did extraction tests with an organic phase made up of 40% N235 and 15% TBP by volume in sulfonated kerosene. The working conditions were set at a 1:1 ratio of organic to aqueous phase, a temperature of 20 °C, an equilibration time of 5 min, and a 6:1 ratio of chloride to cadmium. Figure 6a shows how the starting pH affects the extraction efficiencies of Cd, As, and Zn. As the concentration of hydrogen ions went down, the extraction efficiency of Cd went down as well, just like it did in synthetic solutions. A Cd extraction efficiency of 79.6% was achieved at an initial pH of 0.5.

On the other hand, the extraction efficiency of As was seen to drop at first when acidity decreased and then level off at about 10%. This behavior was ascribed to the prevalence of neutral H_3_AsO_3_ species in low-acidity and high-chloride environments, with only a negligible portion being transformed into extractable anionic complexes [69]. So, the separation factor *β*_Cd/As_ reached a maximum value of 12.7 at pH 0.5. This means that Cd is much more likely to be selected than As. The extraction efficiency of Zn, on the other hand, stayed close to 42%, which is a little lower than what was seen in the synthetic system. This small drop was probably driven by competing extraction effects generated by small amounts of contaminants in the industrial leachate. At pH 0.5, the separation factor *β*_Cd/Zn_ reached 4.44, which meant that considerable separation had been achieved. However, some Zn co-extraction was found to be unavoidable. So, the pH of 0.5 was kept for all the tests that came after.

#### 3.3.2. Effect of Extractant Concentration

Given the complex matrix of the industrial leachate, the impact of N235 concentration on extraction selectivity was systematically evaluated. The corresponding results are depicted in Figure 6b. As the N235 concentration was increased, the extraction efficiencies of Cd and Zn, together with the separation factors *β*_Cd/Zn_ and *β*_Cd/As_, were observed to undergo an initial increase followed by a subsequent decline. At an N235 concentration of 30% by volume, the extraction efficiencies of Cd and Zn were found to reach their respective maxima of 80.7% and 61.1%. Concurrently, *β*_Cd/As_ attained a peak value of 38.8, and *β*_Cd/Zn_ was determined to be 2.66. These values are considered to reflect a robust separation of Cd from As, despite the moderate co-extraction of Zn that was noted.

Although a further increase in N235 concentration to 40% by volume was shown to yield a comparable Cd extraction efficiency (79.6%), it was also observed to undesirably elevate the co-extraction of As to its maximum recorded value of 23.6%. A subsequent increase in extractant concentration to 50% by volume was found to diminish the overall extraction efficiencies. This observation was consistent with the earlier finding described in Section 3.1.2, wherein excessive extractant loading was demonstrated to markedly increase organic phase viscosity and consequently to impede interfacial mass transfer. To minimize reagent consumption while maximizing the separation of Cd from As, an N235 concentration of 30% by volume was deemed optimal for the treatment of the industrial leachate.

#### 3.3.3. Three-Stage Countercurrent Extraction

The results obtained from single-stage extraction experiments indicated that a maximum Cd extraction efficiency of approximately 80% could be achieved. This plateau was recognized as being insufficient to meet the stringent requirements for industrial Cd recovery, a limitation that was primarily ascribed to the competitive binding of abundant Zn(II) ions and other impurity species for the available active extractant sites.

To achieve a deeper recovery of Cd, a three-stage countercurrent extraction simulation was performed under the optimized conditions established in the preceding sections. Specifically, an organic phase consisting of 30% N235 and 15% TBP in sulfonated kerosene was employed, together with an initial pH of 0.5, a chloride-to-cadmium molar ratio of 6 to 1, an organic-to-aqueous phase ratio of 1 to 1, and a temperature of 20 °C. As detailed in Table 2, the multi-stage countercurrent configuration was found to effectively drive the extraction equilibrium in the forward direction, thereby enabling the selective enrichment of the vast majority of cadmium into the organic phase. The cumulative extraction efficiency for Cd was remarkably elevated to 99.80%, whereas the co-extraction of Zn was constrained to 67.6%. Most notably, the co-extraction of As was severely suppressed, declining to a mere 2.19%. These findings unequivocally demonstrate that multi-stage countercurrent extraction is capable of overcoming interference from co-existing impurities, thereby facilitating the profound separation and highly efficient recovery of Cd from complex industrial leachates.

### 3.4. Study on the Loaded Organic Phase Stripping of Cd

#### 3.4.1. Selection of the Stripping Agent

**Figure 7:**
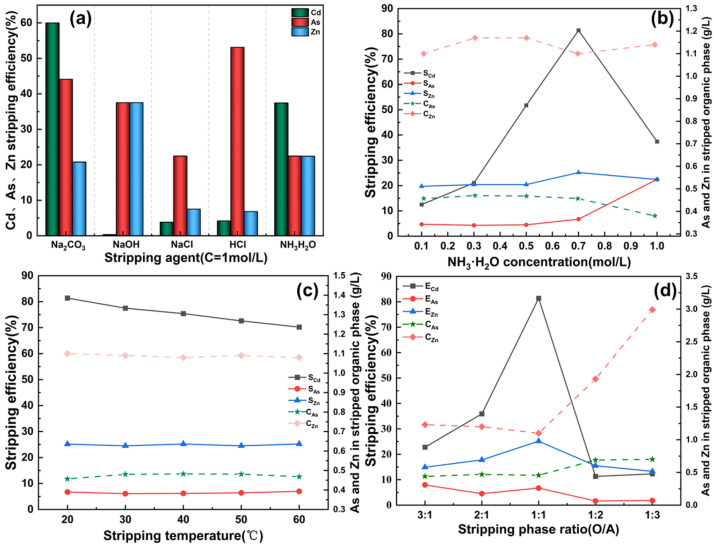
Effects of various parameters on the stripping of Cd: (**a**) stripping agents; (**b**) NH_3_·H_2_O concentration; (**c**) temperature; (**d**) (O/A)phase ratio.

The loaded organic phase obtained from the preceding extraction stage was determined to contain 34.92 g/L Cd, 0.49 g/L As, and 1.47 g/L Zn. Effective stripping was recognized to require the reversal of the extraction equilibrium without compromising the structural integrity of the organic solvent. Various stripping agents, each prepared at a concentration of 1.0 mol/L and including HCl, NaCl, NaOH, NH_3_·H_2_O, and Na_2_CO_3_, were evaluated at an organic-to-aqueous phase ratio of 1 to 1 and a temperature of 20 °C. The corresponding results are presented in Figure 7a.

Although the highest preliminary Cd stripping efficiency of 59.99% was achieved with Na_2_CO_3_, its use was accompanied by the precipitation of CdCO_3_ in the aqueous phase and by severe emulsification of the organic phase. These deleterious effects rendered the system industrially unviable owing to impaired phase disengagement. NaOH was found to be ineffective for Cd stripping, and both acidic and neutral chloride salts (HCl and NaCl) exhibited negligible Cd stripping efficiencies (less than 7%), a consequence of the pronounced stability of the extracted cadmium–chloro complexes. In marked contrast, aqueous ammonia (NH_3_·H_2_O) was observed to successfully strip 37.4% of the loaded Cd in a single stage. This performance was attributed to the formation of highly stable cadmium ammine complexes, specifically Cd(NH_3_)_2_^2+^, while excellent phase separation was concurrently maintained. Accordingly, NH_3_·H_2_O was selected as the optimal stripping agent. The principal stripping reaction is described by the conversion of the extracted complex (R_3_NH)_2_CdCl_4_ with ammonia to yield Cd(NH3)_2_Cl_2_, R_3_NHCl, and water.

(R_3_NH)_2_CdCl_4_ + 2NH_3_·H_2_O = Cd(NH_3_)_2_Cl_2_ + 2R_3_NHCl + 2H_2_O (5)

#### 3.4.2. Optimization of Stripping Parameters

The influence of NH_3_·H_2_O concentration on stripping performance is illustrated in Figure 7b. As the concentration was varied from 0.1 to 1.0 mol/L, the Cd stripping efficiency was found to reach a maximum value of 81.4% at a concentration of 0.7 mol/L. A further increase in concentration to 1.0 mol/L was observed to induce emulsification of the organic phase, which in turn disrupted the functional groups and led to a pronounced decline in stripping efficiency. Under the optimal concentration of 0.7 mol/L, the co-stripping efficiencies of As and Zn were constrained to approximately 5% and 20%, respectively.

The effect of temperature was evaluated over the range of 20 to 60 °C, as depicted in Figure 7c. A slight suppression of Cd stripping was noted with increasing temperature, an observation that is indicative of the exothermic nature of the stripping process. Consequently, ambient temperature (20 °C) was favored for reasons of operational economy and process efficiency.

Furthermore, the organic-to-aqueous phase ratio was found to exert a profound influence on stripping dynamics (Figure 7d). A reduction in the O/A ratio from 3 to 1 to 1 to 1 resulted in a steady increase in Cd stripping, culminating in a maximum value of 81.4%. However, operation at O/A ratios below 1 to 1 was observed to cause a drastic reduction in stripping efficiency to approximately 10%. This decline was primarily ascribed to the excessive accumulation of NH_4_^+^ ions in the aqueous phase, which altered the ionic strength and provoked severe emulsification.

#### 3.4.3. Regeneration of the Organic Phase

Following the ammonia stripping procedure, the stripped organic phase was subjected to washing with 1.0 mol/L HCl to ensure complete regeneration. The residual metal concentrations in the washed organic phase were determined to be negligible (Cd 0.68 g/L, As 0.41 g/L, Zn 1.03 g/L), with values below 1.1 g/L recorded for all metal species. Crucially, this acidic washing step was recognized to facilitate the reprotonation of the tertiary amine groups, wherein R_3_N is converted to R_3_NH^+^, thereby restoring the extraction capacity of the N235 solvent for subsequent continuous recycling operations.

#### 3.4.4. Element Distribution and Equilibrium Analysis of Extraction Process

Based on the conditional experimental results of the extraction, stripping, and acid washing processes described above, a full-process experiment was conducted using high-cadmium smelting dust leachate under optimal conditions. The experiment consisted of three-stage countercurrent extraction, one-stage stripping, and one-stage acid washing. The distribution and mass balance of Cd, As, and Zn throughout the entire process were analyzed, and the results are presented in Table 3. The stripping solution was found to contain 35.23 g/L Cd, 0.033 g/L As, and 0.37 g/L Zn, while the HCl washing solution contained 7.03 g/L Cd, 9.2 mg/L As, and 1.3 mg/L Zn. The overall cadmium recovery efficiency was calculated to be 79.08%, and the purity of the recovered cadmium was 98.9%.

The extractant has extremely low water solubility, resulting in minimal solvent loss due to entrainment and dissolution during extraction. Recovering 1 kg of Cd from the loaded organic phase consumes 1.37 L of NH_3_·H_2_O (~0.94 CNY), and regeneration of the organic phase consumes 2.37 L of HCl (~0.60 CNY), with an additional ~0.2 kg of Cd recovered during the regeneration process.

## 4. Conclusions

This study successfully developed a highly efficient and selective solvent extraction process using the N235/TBP system to recover highly concentrated cadmium from zinc smelting dust leachate. The primary findings are as follows:

An optimal extraction system consisting of 30% N235 and 15% TBP by volume in sulfonated kerosene was established. Under the optimized conditions of an initial pH of 0.5, a chloride-to-cadmium molar ratio of 6 to 1, an organic-to-aqueous phase ratio of 1 to 1, a temperature of 20 °C, and an equilibration time of 5 min, a three-stage countercurrent extraction configuration was shown to extract 99.80% of the cadmium from the highly concentrated industrial leachate. The system was found to effectively reject arsenic, with only 2.19% co-extraction being recorded, thereby achieving a preliminary yet robust separation of Cd from the complex leachate matrix.

FT-IR and ESI-MS analyses unequivocally confirmed that cadmium extraction proceeded via an anion-exchange mechanism. Within the chloride-rich acidic environment, the protonated amine cations R_3_NH^+^ were found to selectively capture the dominant CdCl_4_^2−^ complex. This selectivity was attributed to the favorable thermochemical radius and the relatively weak hydration shell of the CdCl_4_^2−^ anion, which together facilitated the formation of a stable lipophilic complex structurally identified as (R_3_NH)_2_CdCl_4_.

Aqueous ammonia (NH_3_·H_2_O) was proven to be an excellent stripping agent through the formation of stable cadmium ammine complexes without the induction of precipitation. Using 0.7 mol/L NH_3_·H_2_O at an organic-to-aqueous phase ratio of 1 to 1 and a temperature of 20 °C, a single-stage Cd stripping efficiency of 81.4% was achieved. A subsequent washing step with dilute HCl was confirmed to guarantee the complete recovery of residual metals and the structural regeneration of the organic solvent, thereby enabling continuous industrial application.

## Figures and Tables

**Table 1:** Evaluation of precipitation and extraction methods for cadmium recovery.

Precipitation					
Method	Cd conc	Precipitant	Condition	Efficiency	References
Sulfide precipitation	200 mg/L	NaSH	S:Cd^2+^ = 30:1 T = 25 °C [H_3_PO_4_] = 25%H_3_PO_4_	90.34%	[28]
	0.63 g/L	Na_2_S	Na_2_S = 3 g/L t = 60 min;T = 70 °C r = 250 r/min	>99%	[29]
Hydroxide precipitation	1.2 g/L	Ca(OH)_2_	pH = 10 t = 15 min 10% Ca(OH)_2_	97%	[30]
Carbonate precipitation	/	CO_2_	pH = 8 t = 1 h T = 60 °C Q= 50 mL/min	99.69%	[31]
Extraction					
System		Extractant			
Acidic	2.4 g/L	Cyanex 301	O/A = 1:1 pH = 0.3 0.06 M Cyanex301	99.5	[32]
Neutral	0.056 g/L	TBP	O/A = 1:1 pH = 1.5 TBP = 20 vol%	/	[33]
Synergistic extraction	53.5 mg/L	Mextral V10 + Mextral 622H	5% Mextral V10 + 5% Mextral 622H A/O = 1.5:1 pH = 6.5	99.6%	[34]

**Table 2:** Experimental results of three-stage countercurrent extraction of cadmium, arsenic and zinc.

Extraction Raffinate Composition (g/L)				Extraction Stages	Extraction Efficiency (%)		
	Cd	As	Zn		Cd	As	Zn
1	0.32	5.28	0.97	3	99.23	1.6	50
2	0.28	5.26	0.59	3	99.30	2	69.7
3	0.092	5.25	0.63	3	99.80	2.19	67.6

**Table 3:** Distribution and mass balance of elements (Cd, As, Zn) throughout the three-stage countercurrent extraction, one-stage stripping, and one-stage acid washing process.

Input (g/L)		Output (g/L)					Absolute Error (g/L)	Relative Error (%)	Excluding Element Recovery/Removal Rates in the Acid Washing Step (%)
Element	Dust Leaching Solution	Raffinate	Stripped Solution	Regenerated Organic Phase	Acid Washing Solution	Total			
Cd	44.55	0.097	35.23	0.72	7.03	43.08	1.47	3.30	79.08
As	5.77	5.25	0.033	0.44	0.0092	5.73	0.04	0.69	99.43
Zn	2.09	0.52	0.37	1.09	0.0013	1.76	0.11	5.26	82.30

## Data Availability

The original contributions presented in this study are included in the article. Further inquiries can be directed to the corresponding author.

## References

[1] Yu Y., Wang D.H., Yu F., Wang W., Liu S.H., Li D.X. (2024). Current status in the exploration, development, and utilization of cadmium resources in China. China Min. Mag..

[2] Yu D.B., Wang J.P., Xu L., Li D.M. (2015). Analysis of the current situation of cadmium resources in China and suggestions for sustainable development. China Min. Mag..

[3] Wen H.J., Zhu C.W., Du S.J., Fan Y., Luo C.H. (2020). Gallium, germanium, thallium and cadmium resources in China. Chin. Sci. Bull..

[4] Jha M.K., Kumar V., Jeong J., Lee J.C. (2012). Review on solvent extraction of cadmium from various solutions. Hydrometallurgy.

[5] Yan L., Fan Y.U., Huang J., Zhou T.F. (2024). Cadmium metal and its advantages and disadvantages. Bull. Mineral. Petrol. Geochem..

[6] Biswas A., Hendry M.J., Essilfie-Dughan J., Day S., Villeneuve S.A. (2022). Geochemistry of zinc and cadmium in coal waste rock, Elk Valley, British Columbia, Canada. Appl. Geochem..

[7] Yan L., Fan Y., Huang J., Zuo T., Lan B.Y. (2024). Study on the occurrence state and enrichment mechanism of cadmium in sphalerite in Xiwan lead-zinc deposit in Lufang Basin, Anhui Province. Acta Petrol. Sin..

[8] Zhou A., Zhang L., Zhou Y., Li Y.B., Wu X.S., Xia L.G. (2023). Co-smelting process of Pb concentrate and Zn leaching residues with oxygen-rich side blowing furnaces: Industrial application and material balance. JOM.

[9] Ke J.J., Qiu R.Y., Chen C.Y. (1984). Recovery of metal values from copper smelter flue dust. Hydrometallurgy.

[10] Pusateri J.F., Bounds C.O., Lherbier L.W. (1988). Zinc recovery via the reactor process. JOM.

[11] Han G., Wang J., Sun H., Liu B.B., Huang Y.F. (2022). A critical review on the removal and recovery of hazardous Cd from Cd-containing secondary resources in Cu-Pb-Zn smelting processes. Metals.

[12] Xing W., Liu H., Banet T., Wang H.S., Ippolito J.A., Li L.P. (2020). Cadmium, copper, lead and zinc accumulation in wild plant species near a lead smelter. Ecotoxicol. Environ. Saf..

[13] Rudnik E., Nikiel M. (2007). Hydrometallurgical recovery of cadmium and nickel from spent Ni–Cd batteries. Hydrometallurgy.

[14] Hung Y.Y., Yin L.T., Wang J.W., Wang C.T., Tsai C.H., Kuo Y.M. (2018). Recycling of spent nickel–cadmium battery using a thermal separation process. Environ. Prog. Sustain. Energy.

[15] Hazotte C., Leclerc N., Meux E., Lapicque F. (2016). Direct recovery of cadmium and nickel from Ni-Cd spent batteries by electroassisted leaching and electrodeposition in a single-cell process. Hydrometallurgy.

[16] Freitas M., Rosalém S.F. (2005). Electrochemical recovery of cadmium from spent Ni–Cd batteries. J. Power Sources.

[17] Werner T.T., Bell C., Frenzel M., Jowitt S.M., Agarwal P., Mudd G.M. (2024). Cadmium: A global assessment of mineral resources, extraction, and indicators of mine toxicity potential. Environ. Res. Lett..

[18] Godt J., Scheidig F., Grosse-Siestrup C., Esche V., Brandenburg P., Reich A., Groneberg D.A. (2006). The toxicity of cadmium and resulting hazards for human health. J. Occup. Med. Toxicol..

[19] Blumbergs E., Serga V., Platacis E., Maiorov M., Shishkin A. (2021). Cadmium recovery from spent Ni-Cd batteries: A brief review. Metals.

[20] Safarzadeh M.S., Bafghi M.S., Moradkhani D., Ilkhchi M.O. (2007). A review on hydrometallurgical extraction and recovery of cadmium from various resources. Miner. Eng..

[21] Bartolozzi M., Braccini G., Bonvini S., Bonvini S., Marconi P.F. (1995). Hydrometallurgical recovery process for nickel-cadmium spent batteries. J. Power Sources.

[22] Xu Y., Xia H., Zhang Q., Cai W.C., Jiang G.Y., Zhang L.B. (2022). Green and efficient recovery of valuable metals from by-products of zinc hydrometallurgy and reducing toxicity. J. Clean. Prod..

[23] Liu H.L., Wang S.X., Fu L.L., Zhang G.G., Zou Y.G., Zhang L.B. (2022). Mechanism and kinetics analysis of valuable metals leaching from copper-cadmium slag assisted by ultrasound cavitation. J. Clean. Prod..

[24] Córdoba P. (2025). Integrated valorisation of a solid impurity stream in copper production: Recovery and sustainability in metal extraction. Resour. Conserv. Recycl..

[25] Chang Y.L. (2013). Research progress on treatment technologies of cadmium-containing wastewater. Water Purif. Technol..

[26] Parhi P.K., Das N.N., Sarangi K. (2009). Extraction of cadmium from dilute solution using supported liquid membrane. J. Hazard. Mater..

[27] Jha M.K., Kumar V., Singh R.J. (2002). Solvent extraction of zinc from chloride solutions. Solvent Extr. Ion Exch..

[28] Kouzbour S., Gourich B., Gros F., Vial C., Stiriba Y. (2022). A novel approach for removing cadmium from synthetic wet phosphoric acid using sulfide precipitation process operating in batch and continuous modes. Miner. Eng..

[29] Ye Y.H., Lu X.W., Liu Y., Wang Z.R., Ma S.P. (2026). Research on the new high-efficiency technology for removing copper and cadmium in the purification process of zinc hydrometallurgy. Met. Mater. Metall. Eng..

[30] Fu Z.T., Huang W.S., Zheng L.Z. (2010). Study on treatment of cadmium-containing wastewater from Huludao zinc plant by chemical precipitation method. Environ. Prot. Circ. Econ..

[31] Zou F.K., Wang H.B., Xie K., Yang B.W., Deng T., Yang Z.X., Weng Q. (2026). Recovery of tellurium and cadmium from cadmium telluride powder by oxidative leaching. Nonferrous Met..

[32] Reddy B.R., Priya D.N., Park K.H. (2006). Separation and recovery of cadmium (II), cobalt (II) and nickel (II) from sulphate leach liquors of spent Ni–Cd batteries using phosphorus based extractants. Sep. Purif. Technol..

[33] Mellah A., Benachour D. (2007). The solvent extraction of zinc, cadmium and chromium from phosphoric acid solutions by tri-n butyl phosphate in kerosene diluent. Sep. Purif. Technol..

[34] Li X.H., Ai X.B., He L., Ji S.S., Hu M., Ding J.N., Li F. (2017). Solvent extraction separate of zinc and cadmium from magnesium and calcium in sulfuric acid medium by mixing extractants. J. Cent. South Univ..

[35] Xie K., Wen J.K., Hua Y.X., Ruan R. (2008). Selective separation of Cu (II), Zn (II), and Cd (II) by solvent extraction. Rare Met..

[36] Reddy B.R., Priya D.N., Kumar J.R. (2004). Solvent extraction of cadmium (II) from sulphate solutions using TOPS 99, PC 88A, Cyanex 272 and their mixtures. Hydrometallurgy.

[37] Daud H., Cattrall R.W. (1981). The extraction of Hg (II) from potassium iodide solutions and the extraction of Cu (II), Zn (II) and Cd (II) from hydrochloric acid solutions by Aliquat 336 dissolved in chloroform. J. Inorg. Nucl. Chem..

[38] Owusu G. (1998). Selective extractions of Zn and Cd from Zn, Cd, Co, Ni sulphate solution using di-2-ethylhexyl phosphoric acid extractant. Hydrometallurgy.

[39] Tian M., Mu F., Jia Q., Quan X.J., Liao W.P. (2011). Solvent extraction studies of zinc (II) and cadmium (II) from a chloride medium with mixtures of neutral organophosphorus extractants and amine extractants. J. Chem. Eng. Data.

[40] Gupta B., Deep A., Malik P. (2001). Extraction and recovery of cadmium using Cyanex 923. Hydrometallurgy.

[41] Almela A., Elizalde M.P., Gómez J.M. (1998). Cadmium (II) extraction from phosphoric media by bis (2, 4, 4-trimethylpentyl) thiophosphinic acid (Cyanex 302). Fluid Phase Equilibria.

[42] El Dessouky S.I., El-Nadi Y.A., Ahmed I.M., Saad E.A., Daoud J.A. (2008). Solvent extraction separation of Zn (II), Fe (II), Fe (III) and Cd (II) using tributylphosphate and Cyanex 921 in kerosene from chloride medium. Chem. Eng. Process. Process Intensif..

[43] Fatmehsari D.H., Darvishi D., Etemadi S., Hollagh A.E., Alamdari E.K. (2009). Interaction between TBP and D2EHPA during Zn, Cd, Mn, Cu, Co and Ni solvent extraction: A thermodynamic and empirical approach. Hydrometallurgy.

[44] Kumar V., Kumar M., Jha M.K., Jeong J., Lee J.C. (2009). Solvent extraction of cadmium from sulfate solution with di-(2-ethylhexyl) phosphoric acid diluted in kerosene. Hydrometallurgy.

[45] Leopold A.A., Coll M.T., Fortuny A., Rathore N.S., Sastre A.M. (2010). Mathematical modeling of cadmium (II) solvent extraction from neutral and acidic chloride media using Cyanex 923 extractant as a metal carrier. J. Hazard. Mater..

[46] Parus A., Wieszczycka K., Olszanowski A. (2011). Solvent extraction of cadmium (II) from chloride solutions by pyridyl ketoximes. Hydrometallurgy.

[47] Reddy B.R., Priya D.N. (2006). Chloride leaching and solvent extraction of cadmium, cobalt and nickel from spent nickel–cadmium, batteries using Cyanex 923 and 272. J. Power Sources.

[48] Reddy B.R., Rao S.V., Priya D.N. (2008). Selective separation and recovery of divalent Cd and Ni from sulphate solutions with mixtures of TOPS 99 and Cyanex 471 X. Sep. Purif. Technol..

[49] Qian Z., Li C., He L., Chen H.F., Peng Y.M., Shi Q.L., Xiong X.L., Xia X.B., Wang C.H. (2024). Efficient separation of cadmium from cobalt-rich solutions using cyanex301 and P204/P507. J. Sustain. Metall..

[50] Singh O.V., Tandon S.N. (1975). Extraction of cadmium as chloride by high molecular weight amines and quaternary ammonium salt. J. Inorg. Nucl. Chem..

[51] Sato T., Shimomura T., Murakami S., Maeda T., Nakamura T. (1984). Liquid-liquid extraction of divalent manganese, cobalt, copper, zinc and cadmium from aqueous chloride solutions by tricaprylmethylammonium chloride. Hydrometallurgy.

[52] He Y., Li Y.W., Li S.W., Yin S.H., Zhang L.B. (2023). Purification of chlorine-containing copper smelting wastewater using extraction-stripping-salting out method. React. Chem. Eng..

[53] Wassink B., Dreisinger D., Howard J. (2000). Solvent extraction separation of zinc and cadmium from nickel and cobalt using Aliquat 336, a strong base anion exchanger, in the chloride and thiocyanate forms. Hydrometallurgy.

[54] Zhang T., Jiang T., Liu Z. (2019). Recovery of Ge (IV) from synthetic leaching solution of secondary zinc oxide by solvent extraction using tertiary amine (N235) as extractant and trioctyl phosphate (TOP) as modifier. Miner. Eng..

[55] Wang P.C., Liu Z.H., Zhang T., Liu Z.Y., Zhu D.P., Jiang T. (2023). Extraction mechanism of germanium in sulfate solutions using a tertiary amine (N235)-based solvent extraction system. Sep. Purif. Technol..

[56] Xie Q.Y., Chen J., Yang X.J., Wang Y. (2007). Mechanism of TBP on Eliminating Third Phase in N235-HCl Extraction System. Chin. J. Inorg. Chem..

[57] Berthon L., Paquet A., Saint-Louis G., Guilbaud P. (2021). How phase modifiers disrupt third-phase formation in solvent extraction solutions. Solvent Extr. Ion Exch..

[58] Duan W.J., Wang Y.Y., Li R.J., Ren Z.Q., Zhou Z.Y. (2024). Selective extraction of lithium from high magnesium/lithium ratio brines with a TBP–FeCl3–P204–kerosene extraction system. Sep. Purif. Technol..

[59] Xu C.X., Zhou J.W., Yin S.H., Zhang L.J., Hu S.X., Li X., Li S.W. (2021). Solvent extraction and separation of zinc-iron from spent pickling solution with tri-n-octylamine. Sep. Purif. Technol..

[60] Tomaszewska M., Borowiak-Resterna A., Olszanowski A. (2007). Cadmium extraction from chloride solutions with model N-alkyl-and N, N-dialkyl-pyridine-carboxamides. Hydrometallurgy.

[61] Yang X.W., Qiu D.F. (2011). Hydrometallurgy.

[62] Xie H.J., Jiang N., Guo X.Z., Liu Z.Z., Cheng W. (2024). Adsorption performance of magnetic sodium lignosulfonate hydrogel for crystal violet in aqueous solutions. Chin. J. Environ. Eng..

[63] Nyamato G.S., Wambugu K., Kiratu J., Ojwach S.O. (2022). Liquid-liquid extraction of copper (II), zinc (II), cadmium (II), and lead (II) from aqueous solution and sewage effluent using phenoxy-amino ligands. Water Sci. Technol..

[64] Lommelen R., Binnemans K. (2021). Hard–soft interactions in solvent extraction with basic extractants: Comparing zinc and cadmium halides. Acs Omega.

[65] Qin Z.F., Zhang G., Xiong Y., Luo D.M., Li C., Tang S.Y., Yue H.Y., Liang B. (2020). Recovery of vanadium from leach solutions of vanadium slag using solvent extraction with N235. Hydrometallurgy.

[66] Chen F., Wang X., Liu W., Liang B., Yue H.R., Li C. (2016). Selective extraction of nitric and acetic acids from etching waste acid using N235 and MIBK mixtures. Sep. Purif. Technol..

[67] Ma R.J. (2009). Solvent Extraction Metallurgy.

[68] Sato T., Adachi K., Kato T., Nakamura T. (1982). The extraction of divalent manganese, cobalt, copper, zinc, and cadmium from hydrochloric acid solutions by tri-n-octylamine. Sep. Sci. Technol..

[69] Ten M., Liang L.N., Cai Y.Q., Mou S.F., Guo W., Zhu Y.G., Jiang G.B. (2007). Hyphenation of ion chromatography and hydride generation atomic fluorescence spectrometry for arsenic speciation. Chin. J. Anal. Lab..

